# Early-onset obsessive-compulsive disorder: sociodemographic and clinical characterization of a large outpatient cohort

**DOI:** 10.1192/j.eurpsy.2024.141

**Published:** 2024-08-27

**Authors:** B. Benatti, N. Girone, M. Vismara, C. Bucca, B. Dell’Osso

**Affiliations:** ^1^Department of Mental Health, Sacco University Hospital; ^2^Center for Neurotechnology and Brain Therapeutic, Aldo Ravelli Center, University of Milan, Milan, Italy; ^3^Department of Psychiatry and Behavioral Sciences, Bipolar Disorders Clinic, Stanford University, Stanford, United States

## Abstract

**Introduction:**

Obsessive-compulsive disorder (OCD) is a prevalent and disabling condition characterized by a wide variety of phenotypic expressions. Several studies have reinforced the hypothesis of OCD heterogeneity by proposing subtypes based on predominant symptomatology (Mataix-Cols et al., 2005), course (Tukel et al., 2007), and comorbidities (Mahasuar et al., 2011). Early-onset OCD could be considered a neurodevelopmental subtype of OCD, with evidence of distinct neurocircuits supporting disease progression (Park et al., 2022).

**Objectives:**

The aim of the present study is to evaluate the sociodemographic and clinical differences between the early-onset and late-onset subtypes in a large patient cohort.

**Methods:**

Two hundred and eighty patients diagnosed with OCD were consecutively recruited from the OCD Tertiary Clinic at Luigi Sacco University Hospital in Milan. Sociodemographic and clinical variables were analyzed for the entire sample and compared between the two subgroups (EO: early-onset, age <18 years [40%]; LO: late-onset, age ≥ 18 years [60%]).

**Results:**

The EO group showed a higher frequency of male gender (65.5% vs 34.5%, p< .001, see Figure 1a), a higher presence of lifetime psychiatric comorbidities (75.7% vs 24.3%, p =.025), and higher rates of Tic and Tourette disorders (7.2% vs 0%, p=.006) compared to the LO group. Additionally, in the EO subgroup, a longer duration of untreated illness was observed (9.05 ± 10.0 vs 5 ± 7.17; p<.001, see Figure 1b), along with a lower presence of insight (33.3% vs. 66.7%, p =.024). No significant differences emerged in the Yale-Brown Obsessive-Compulsive Scale scores between the groups.

**Image:**

**Figure:**
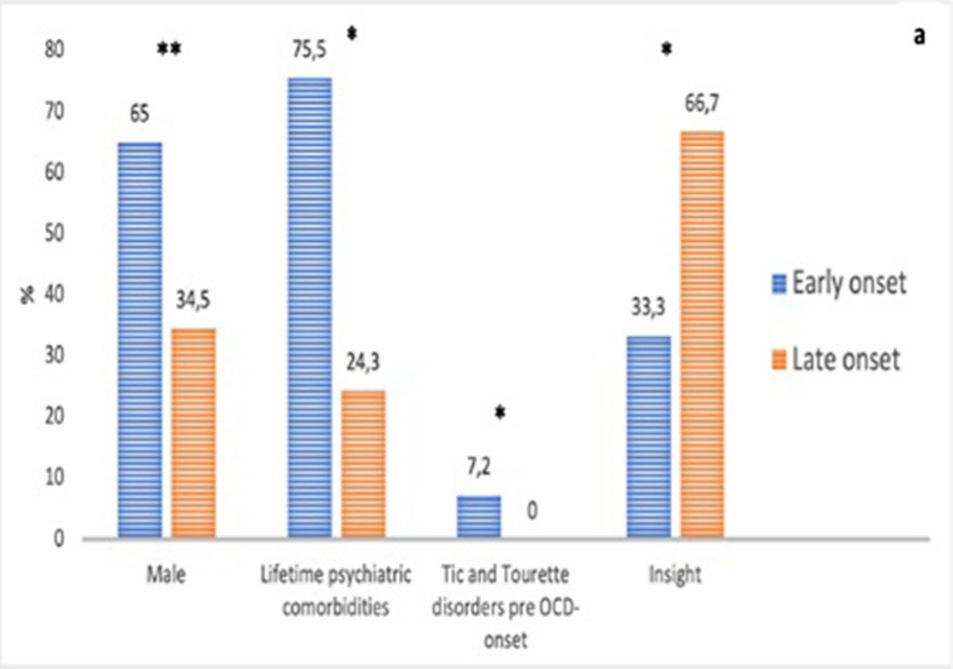

**Image 2:**

**Figure:**
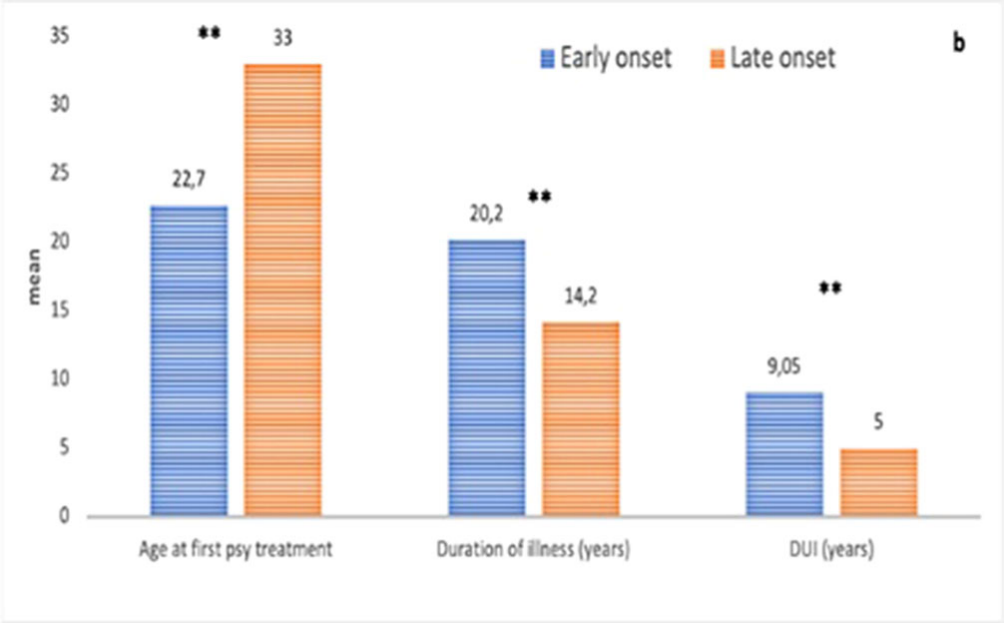

**Conclusions:**

The early-onset OCD subtype highlights a more severe psychopathological profile compared to the late-onset group. Exploring distinct manifestations and developmental trajectories of OCD can contribute to a better definition of homogeneous subtypes, useful for studying risk factors and defining targeted therapeutic strategies for treatment.

**Disclosure of Interest:**

B. Benatti Speakers bureau of: Angelini, Lundbeck, Janssen, Rovi, N. Girone: None Declared, M. Vismara: None Declared, C. Bucca: None Declared, B. Dell’Osso Grant / Research support from: Angelini, Lundbeck, Janssen, Pfizer, Otzuka, Neuraxpharm, and Livanova, Speakers bureau of: Angelini, Lundbeck, Janssen, Pfizer, Otzuka, Neuraxpharm, and Livanova.

